# The effects of lifestyle change on indicators of cardiometabolic health in semi-nomadic pastoralists

**DOI:** 10.1093/emph/eoad030

**Published:** 2023-09-25

**Authors:** Zane S Swanson, Hilary Bethancourt, Rosemary Nzunza, Emmanuel Ndiema, David R Braun, Asher Y Rosinger, Herman Pontzer

**Affiliations:** Department of Evolutionary Anthropology, Duke University, Durham, NC, USA; Department of Anthropology, Northwestern University, Evanston, IL, USA; Kenya Medical Research Institute (KEMRI), Nairobi, Kenya; Department of Earth Sciences, National Museums of Kenya, Nairobi, Kenya; Center for the Advanced Study of Human Paleobiology, Department of Anthropology, The George Washington University, Washington, DC, USA; Technological Primates Research Group, Max Planck Institute for Evolutionary Anthropology, Deutscher Platz 6, 04103, Germany; Department of Anthropology, Pennsylvania State University, University Park, PA, USA; Department of Biobehavioral Health, Pennsylvania State University, University Park, State College, PA, USA; Department of Evolutionary Anthropology, Duke University, Durham, NC, USA; Duke Global Health Institute, Duke University, Durham, NC, USA

**Keywords:** noncommunicable disease, pastoralism, market integration, sedentarization, evolutionary mismatch

## Abstract

**Background and objectives:**

Non-communicable disease risk and the epidemic of cardiometabolic diseases continue to grow across the expanding industrialized world. Probing the relationships between evolved human physiology and modern socioecological conditions is central to understanding this health crisis. Therefore, we investigated the relationships between increased market access, shifting subsistence patterns and cardiometabolic health indicators within Daasanach semi-nomadic pastoralists who vary in their engagement in traditional lifestyle and emerging market behaviors.

**Methodology:**

We conducted cross-sectional socioecological, demographic and lifestyle stressor surveys along with health, biomarker and nutrition examinations among 225 (51.6% female) Daasanach adults in 2019–2020. We used linear mixed-effects models to test how differing levels of engagement in market integration and traditional subsistence activities related to blood pressure (BP), body composition and blood chemistry.

**Results:**

We found that systolic and diastolic BP, as well as the probability of having high BP (hypertension), were negatively associated with distance to market, a proxy for market integration. Additionally, body composition varied significantly by socioeconomic status (SES), with significant positive associations between BMI and body fat and higher SES among adults.

**Conclusions and implications:**

While evidence for evolutionary mismatch and health variation have been found across a number of populations affected by an urban/rural divide, these results demonstrate the effects of market integration and sedentarization on cardiometabolic health associated with the early stages of lifestyle changes. Our findings provide evidence for the changes in health when small-scale populations begin the processes of sedentarization and market integration that result from myriad market pressures.

## INTRODUCTION

Understanding the relationships between health and lifestyle is of growing global importance. Populations around the world are transitioning toward states of industrialization and urbanization that characterize ‘Western’ lifestyles. This shift has a distinct importance for a rapidly changing Eastern Africa [1–6]. Shifts away from traditional subsistence and movement strategies create potential mismatches between evolved physiology and the ecological and behavioral environments populations create [7–9]. Most commonly, research investigating the repercussions of evolutionary discordance on cardiometabolic health has either focus on inter-population variation between ‘Western’ populations in developed countries and small-scale populations in developing countries or the intra-population variation found within groups that express variation in lifestyle variables (e.g. market integration, urbanization, subsistence strategy) [6–8, 10–15]. The investigation of non-communicable diseases (NCDs), such as cardiometabolic diseases, is central to interpretations of these potential mismatches within the study of evolutionary health. Globally, the prevalence of hypertension is increasing and is the leading risk factor of death globally, accounting for 10.4 million deaths annually [16, 17].

Sedentarization, or the transition from a previously physically active lifestyle to a settled one, has occurred in many communities since the Neolithic Transition, some 10 000 years ago. Increased rates of geographic encroachment by larger settled populations, climate change, governmental intervention or a combination of the three has led to an escalating trend toward sedentary and market-integrated lifestyles among non-industrialized populations [2, 3, 18–22]. Often, NGOs and governmental agencies encourage the transition of nomadic populations to more sedentary ways of life [23]. However, the health outcomes of individuals who have become more settled have often been found to be worse than those of more nomadic individuals in a population [3, 14, 24]. It has been suggested that this disparity in health results from changes in the dietary environment, increased exposure to pathogens due to higher population and waste densities, and lower levels of physical activity. Some of these changes are additionally influenced by increased market access, which has been associated with becoming more settled [14].

The negative implications of sedentarization and lifestyle changes have often been explained by an evolutionary mismatch in which deleterious effects arise among individuals for whom subsistence contexts have rapidly changed away from the one in which their populations have gained biological and behavioral adaptations [2, 9, 25–28]. This process can be understood as an ultimate cause of more proximate factors that can affect human health and life history, such as nutrition, stress and disease. One way to assess the potential life history, health and behavioral consequences of lifestyles in flux is to perform intra-group comparisons with a population currently undergoing this transition.

Work with populations that exhibit substantial lifestyle variation provides insight into the associations between diet, activity and health outcomes, such as hypertension, diabetes, overweight/obesity and heart disease. However, studies that identify intra-population variation in health often do so through the framework of an ‘urban-rural divide’, which precludes the ability to specifically investigate the effects on a population as it actively transitions away from traditional lifestyle characteristics. Here, we work with traditionally semi-nomadic Daasanach pastoralists living in northern Kenya who are experiencing a relatively recent transition toward more sedentary lifestyles in a remote non-urban area to investigate the effects of lifestyle change on cardiometabolic health. Daasanach in northern Kenya provides the unique opportunity to understand the ways in which biobehavioral health variation occurs at a level more finite than the ‘urban-rural divide’, as they have a continuum of variation in household characteristics through distance in community settlements to a main market town. This investigation will both serve to enhance our understanding of the effects of market integration and sedentarization on health variation within a genetically homogenous population in the early stages of lifestyle change and serve to provide additional context for the comparative study of evolutionary health across global populations.

## METHODS

### Ethical approvals

Study protocols were approved by the Penn State Institutional Review Board (IRB#STUDY00009589) and the Kenya Medical Research Institute (#KEMRI/RES/7/3/1) prior to all data collection. These approvals include permissions for the collection of health records from the Illeret Health Clinic, in addition to anthropometric, survey and biological sample collection. In-person consent for data collection was also obtained from all relevant sources, including community elders, the Illeret Ward and medical officials from the IHC. Additionally, permitting for human biology research was obtained from the National Commission for Science, Technology and Innovation (NACOSTI).

### Study population: Daasanach of northern Kenya

**Figure 1.**
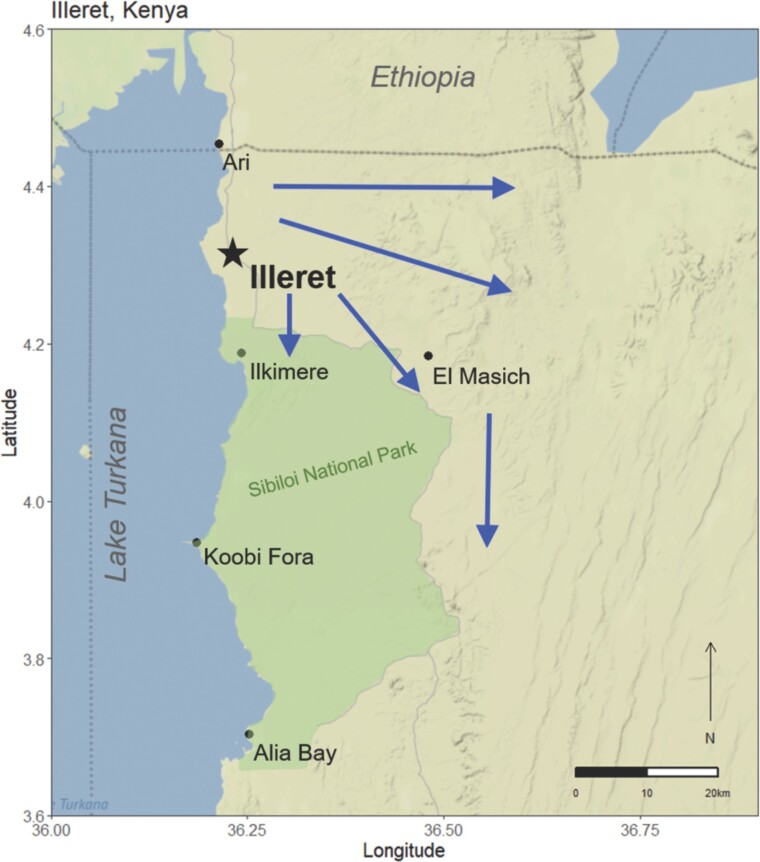
Study area with major landmarks: arrows indicate travel into the fora and away from more developed/settled zones. (Adapted from Mwamidi et al. [31])

Daasanach are a semi-nomadic pastoral group living in semi-arid and arid regions of southwestern Ethiopia and northwestern Kenya (Fig. 1) [29, 30]. Forty-eight thousand Daasanach are reported to live in Ethiopia, with about 19 300 Daasanach living in Kenya according to recent census reporting [31, 32]. Most of the Daasanach population in Kenya live in or around the town of Illeret (4.314° N, 36.227° E), located in the most northwestern region of Marsabit county, bordering Lake Turkana to the west and Ethiopia to the north. The region around Illeret experiences a bimodal seasonal cycle with mean temperatures ranging from 20°C to 37°C and yearly average rainfall of about 217 mm [33, 34]. The increased threat of drought, flash flooding and climate variability, as well as historical economic and political isolation, have led to health issues related to long-term water and food insecurity for Daasanach living in Kenya [35–40].

Traditional Daasanach lifestyles are highly physically demanding. Men are typically in charge of protecting and caring for livestock, with herding responsibilities falling to boys beginning in young adolescence. Women and girls are responsible for most other subsistence labor tasks. These responsibilities include water gathering, childcare, cooking and home building/maintenance. Daasanach also practice polygyny, with men often having multiple wives who typically serve as individual female heads of the household once they have had their first child. Subsistence labor varies as a function of household mobility and engagement in pastoral activity for both men and women.

Though considered semi-nomadic, Daasanach in and around Illeret engage in a variety of movement patterns. Those living in Illeret tend to be more settled, living more often in permanent living structures and with increased access to organizational infrastructure. Daasanach living in the upper Turkana Basin have a heterogenous settlement pattern, with some communities, specifically those closer to the town of Illeret, being more settled than the semi-nomadic communities in surrounding areas [31]. These semi-nomadic and nomadic communities travel into what is called the fora, or undeveloped pastureland to the South and East of Illeret (Fig. 1).

Unlike Daasanach living in the Omo River Valley of Ethiopia, who traditionally practice agropastoralism [29], Daasanach in and around Illeret are far more reliant on herding practices and access to market food staples, such as beans, rice and maize, with very little ability to perform sustainable agriculture. Due to the severity and reciprocal relationship of water and food insecurity in the region, which has recently been measured at the household level across several Daasanach communities, malnutrition is a major concern in the area [39–41]. Though several standpipes can be found in Illeret, retrieving water from hand-dug wells—which can measure nearly 2 m in depth—in dry riverbeds (lagas) is often preferred due to its ease of access, the quality of water and the cost of piped water [39, 41]. This way of collecting water can be very labor intensive, with Daasanach female heads of household reporting multiple daily water collection trips of multiple hours to collect water for household use.

### Data collection

To test the associations between lifestyle variables with indicators of health, anthropometric, survey and GPS data (household, water source, points of interest locations) were collected over the course of two field seasons between June–July of 2019 and February–March of 2020 in the region surrounding Illeret in Marsabit County, Kenya. The summer field season of 2019 was characterized by hot and dry weather typical of the summer dry season. Conversely, the 2020 field season was characterized by increased precipitation prior to the typical spring rainy season. This study took place just before the onset of the severe 2020–2023 drought. Collection took place across 11 communities located between 0.05 km and 32.87 km from the nearest market centers—the Illeret Health Clinic (4.314° N, 36.227° E), the Sieslucho fish market (4.398° N, 36.221° E) and the Ari trading post (4.454° N, 36.213° E) located on the border of Kenya and Ethiopia. Nine of the communities sampled were permanent, whereas two were temporary settlements used by individuals practicing nomadic pastoralism. Such temporary settlements are typically used for several days to several weeks and vary in location based on seasonal and climatic changes [31]. The communities were selected purposively to have variation in distance from the main market town of Illeret and variation in access to water sources. Data from all subjects (*n* = 251; age = 18–79) were collected in an opportunistic but systematic manner, with subjects selected for participation from every third household from a central community point to minimize family clusters. Women who were pregnant and non-Daasanach community members were excluded from analyses, reducing our final sample by 26 participants (*n* = 225; male = 106; female = 119). A subsample (*n* = 55) of adults who participated before noon also had blood chemistry analyzed.

Participation included the collection of anthropometric and cardiometabolic biomarker data in coordination with a semi-structured interview to collect survey data related to lifestyle variables (e.g. labor, mobility and market interaction), socioeconomic status, health, perceived psychosocial stress, demography, food insecurity and water insecurity. Food insecurity was assessed using the 9-item Household Food Insecurity Access Scale, while the validated 12-item Household Water Insecurity Experience Scale was used for quantifying experiences of water access, use and reliability [42, 43] as described elsewhere [40]. All interviews were completed with the help of a local research assistant and translator.

#### Anthropometric outcomes.

- Weight and body fat percentage: Participants had their weight (to the nearest 0.1 kg) and body fat percentage (to the nearest 0.1%) measured using a Tanita BF-680W bioelectronic impedance scale (Tanita; Arlington Heights, IL). Participants were weighed with clothes but asked to remove any excess clothing, hats and jewellry.
- Height: Height was measured without shoes (to the nearest 0.1 cm) using a portable Seca stadiometer, which was placed on a hard and smooth surface.
- Body mass index: BMI was calculated using the subject’s measured weight and height (kg/m^2^) and individuals were classified as underweight if their BMI was <18.5 kg/m^2^ [44].
- Skinfolds: Four skinfold measurements—triceps, biceps, suprailiac and subscapular—were taken using Lange skinfold calipers (to the nearest mm) following standard technique [45].
- Blood pressure (BP): Systolic and diastolic BP was measured twice using an Omron Series 7 upper-arm digital BP monitor (Omron; Kyoto, Japan). Participants were seated for both measurements, with a seated rest of several minutes between each measurement. Analyses were completed with the second measured BP to account for the potential effects of unfamiliarity with the procedure, sometimes referred to as white coat hypertension [46, 47].
- Hypertension was defined as diastolic BP ≥140 and/or systolic BP ≥90) (also known as stage 2 hypertension) [47].
- Blood chemistry: Dried blood spot samples and fingerstick blood samples were also taken concurrently, and the latter were analyzed for blood glucose, triglycerides and cholesterol (total, high-density lipoprotein [HDL] and low-density lipoprotein [LDL]) using a Cardiochek Plus analyzer (PTS Diagnostics; Whitestone, IN, USA). Subjects were asked the timing of their last caloric intake prior to blood chemistry analysis but were not explicitly asked to fast prior to data collection. All blood chemistry data were collected before noon.

#### Primary predictors.

- Distance to market town: We collected GPS coordinates of each household and analyzed the distance to the nearest market center (Illeret Health Clinic, Sieslucho fish market, Ari trading post) as the natural log (in meters) as a proxy for market integration, similar to prior work [6, 11]. Log transformation was used to remove heteroscedasticity.
- Mobility: Household mobility was measured as the square root of the number of household moves in the previous year, a proxy for engagement in traditional nomadic activity.
- Socioeconomic Status or Position (SES): To account for the culturally specific context within which socioeconomic standing could be assessed across Daasanach communities, SES was measured as the standardized value of the first principal component (PC1) score for sex-specific PCAs that included individual measures of reported income, livestock wealth and SES perception.
  ◦ Socioeconomic perception: SES perception was measured by asking household heads to rank themselves on the McArthur ladder from 1 (worst off) to 10 (best off) relative to others in their community regarding income, education and social status [48].
  ◦ Household income: Income was measured by reported income earned in the previous month.
  ◦ Livestock wealth: Livestock wealth was calculated as the product of the number of each type of livestock owned (sheep, goats, cows, camels, donkeys) and the reported market value of each type of livestock in the region ([40] for additional details).

### Data analysis

All statistical analyses were completed in R (4.1.1). To identify the relationships between lifestyle variables, Pearson pairwise correlation matrices were constructed. These models specifically compared variables related to lifestyle behaviors, wealth, experienced food and water insecurity and market integration. Variables tested included: market distance, household mobility, household size, food/water insecurity, water labor, education, perception of socioeconomic status, household income and livestock. These corrections were used to account for heteroscedasticity in the data that occurred as a function of the scale over which these variables varied in the sample. All comparisons were done with Pearson pairwise testing and the significance of observed relationships was established at *P* values less than 0.05.

Linear mixed-effect models, with household identification as a random effect, were used to test for the effects of lifestyle on health across all subjects. Models included age, sex, distance to market, household mobility, household size and SES as fixed effects. Community identification was not used for models with distance to market to eliminate the effect of multicollinearity given the amount of homogeneity in market distance values within communities. All continuous variables were standardized before their inclusion in linear models. When significant sex interactions were detected for variables within the linear mixed effects model, models for males and females were estimated separately. Logistic regression was also used to test the relationship between lifestyle and odds of hypertension in our sample.

## RESULTS

### Market and lifestyle correlates

**Figure 2.**
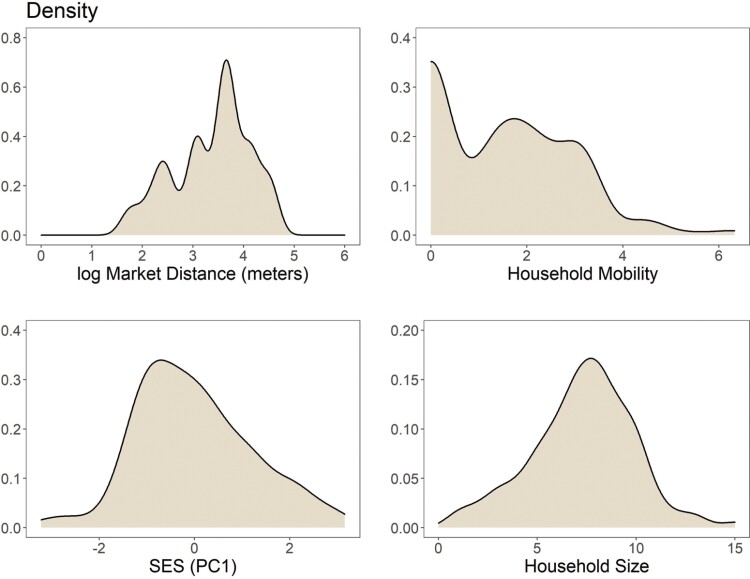
Kernel density representations of distance to the nearest market center (ln meters), household mobility (square root of the reported number of moves in the previous year), population-specific representation of socioeconomic status (PC1 score) and reported household size

**Figure 3.**
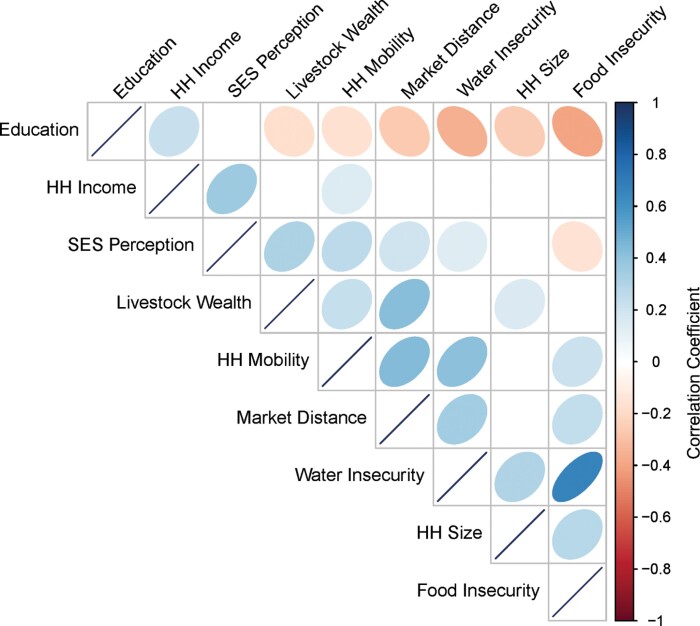
Pearson pairwise correlation matrix of lifestyle, socioeconomic and socioecological measures. Education measured as the reported number of grades completed, household (HH) income calculated as the natural log of reported income in the previous month, socioeconomic status (SES) perception measured at the self-identified placement on a 10-scale SES ladder relative to others in their community, livestock wealth calculated as the natural log of the product of reported livestock ownership and market value of livestock at the time of recording, HH mobility calculated at the square root of reported number of household moves in the previous year, market distance measured as the natural log of meters from subjects’ households to the nearest market center, HH size measured as the reported number of individuals living in the household, and water and food insecurity measured via Household Water Insecurity Experience and Household Food Insecurity Access Scale questionnaires

Household measures of lifestyle, such as market distance, SES and household mobility and size varied across our study sample (Fig. 2). Mean household distance to the nearest market center was 6.8 ± 9.4km, while the mean household size was reported as 7.5 ± 2.7 individuals, and mean household mobility was reported at 4.0 ± 6.1 moves in the previous year. Relationships between lifestyle variables were assessed using Pearson’s pairwise correlation analysis (Fig. 3). Distance to market was found to be significantly positively correlated with household mobility and livestock wealth, as households farther from market centers engage in more traditional semi-nomadic pastoral activities. Market distance and household mobility were both negatively correlated with education. Water and food insecurity were positively correlated with each other. Additionally, both food and water insecurity were positively correlated with household mobility and market distance, with individuals living farther from sources of infrastructure finding themselves at higher risk of undernutrition.

### Age-related variation in cardiometabolic health and body composition

Overall, Daasanach adults maintained relatively low rates of hypertension (men: 26.2%, women: 24.3%) and low BMI values (men = 17.9 ± 2.8 kg/m^2^, women = 18.3 ± 3.1 kg/m^2^) compared to Western populations (Tables 1 and 2). To identify variation across indicators of cardiometabolic health in Daasanach adults, variables related to cardiovascular health and body composition were evaluated across the adult age course. The mean age of males sampled (*n* = 106) was 46.3 ± 15.8 years, while the mean age for females (*n* = 119) was 35.4 ± 12.0 years. Systolic and diastolic BP increased with age (systolic: *β* = 0.32 mm Hg, SE = 0.08, *P* < 0.01 | diastolic: *β* = 0.21 mm Hg, SE = 0.08, *P* < 0.01) among adult Daasanach men and women (Tables 3 and 4; Supplementary Fig. 1).

**Table 1.** Mean and standard deviation values and observed hypertension prevalence among Daasanach adults

Sample (age)	*N*	Systolic BP (mmHg)	Diastolic BP (mmHg)	Hypertensive
Female (18–39)	76	110.7 (±11.5)	80.7 (±9.2)	15 (19.7%)
Female (40+)	39	119.0 (±16.6)	84.9 (±11.2)	13 (33.3%)
Female (total)	115	113.5 (±14.0)	82.1 (±10.1)	28 (24.3%)
Male (18–39)	42	115.3 (±11.8)	80.0 (±7.5)	5 (11.9%)
Male (40+)	61	122.1 (±20.1)	86.0 (±13.1)	22 (36.1%)
Male (total)	103	119.3 (±17.4)	83.6 (±11.5)	27 (26.2%)

Hypertension defined as systolic BP ≥ 140 mmHg and/or diastolic BP ≥ 90 mmHg [47].

**Table 2.** Mean and standard deviations of biomarkers for body composition and prevalence of underweight among Daasanach adults

Sample (age)	*N*	Body fat (%)	BMI (kg/m^2^)	Underweight *n* (%)
Female (18–39)	77	21.3 (±6.7)	18.3 (±2.1)	43 (55.8%)
Female (40+)	42	23.9 (±7.9)	18.3 (±4.4)	25 (59.5%)
Female (total)	119	22.2 (±7.2)	18.3 (±3.1)	68 (57.1%)
Male (18–39)	43	8.3 (±2.0)	17.9 (±1.2)	33 (76.7%)
Male (40+)	63	10.7 (±4.3)	17.9 (±3.5)	45 (71.4%)
Male (total)	106	9.7 (±3.7)	17.9 (±2.8)	78 (73.6%)

Underweight defined as having a BMI ≤ 18.5.

**Table 3.** Linear mixed effect models testing the associations between lifestyle changes and indicators of cardiometabolic health among Daasanach adults

	Dependent variable							
	BMI	Body fat %		Sum of skinfolds		Systolic BP	Diastolic BP	Hypertension
Fixed effects, β (SE)		(female)	(male)	(female)	(male)			
Age	–0.068	**0.240** ^**^	0.079^*^	–0.161	0.045	**0.317** ^***^	**0.207** ^**^	0.495^**^
	(0.068)	**(0.110)**	(0.046)	(0.121)	(0.060)	**(0.080)**	**(0.083)**	(0.238)
Sex	–0.087					0.170	–0.0003	–0.231
	(0.114)					(0.137)	(0.139)	(0.416)
Market distance	–0.075	–0.145^*^	–0.044	–0.084	–0.012	–0.255^***^	–0.284^***^	–0.516^**^
	(0.065)	(0.085)	(0.051)	(0.093)	(0.067)	**(0.076)**	**(0.079)**	**(0.238)**
Household mobility	–0.046	–0.064	–0.063	–0.121	–0.163^***^	0.027	–0.035	–0.134
	(0.064)	(0.086)	(0.047)	(0.096)	**(0.060)**	(0.074)	(0.078)	(0.226)
Household size	–0.011	–0.0005	0.055	–0.090	–0.022	–0.192^***^	–0.038	0.009
	(0.062)	(0.085)	(0.045)	(0.093)	(0.059)	**(0.072)**	(0.075)	(0.211)
SES (PC1 score)	**0.216** ^***^	0.159^*^	**0.101** ^**^	**0.205** ^**^	**0.184** ^***^	0.057	–0.021	–0.060
	**(0.061)**	(0.084)	**(0.044)**	**(0.094)**	**(0.058)**	(0.071)	(0.074)	(0.205)
Observations^a^	221	116	101	118	105	214	214	214
Log likelihood	–267.34	–372.87	–272.22	–490.86	–422.10	–292.11	–297.42	–60.18
Akaike Inf. Crit.	552.68	763.74	560.52	999.72	861.10	602.21	612.85	136.36
Bayesian Inf. Crit.	583.27	788.52	581.44	1024.66	883.23	632.50	643.14	158.25

^*^ *P* < 0.1; ^**^P < 0.05; ^***^P < 0.01.

^a^Missing data due to subjects declining measurement.

**Table 4.** Linear mixed effect models testing the associations between lifestyle changes and blood chemistry indicators of cardiometabolic health among a subsample of Daasanach adults.

Blood chemistry subsample						
	Dependent variable					
	Total cholesterol	HDL		LDL		Triglycerides
Fixed effects, *β* (SE)		(Female)	(Male)	(Female)	(Male)	
Age	0.011	–0.030	–0.177	0.349	–0.323	0.066
	(0.198)	(0.399)	(0.255)	(0.288)	(0.283)	(0.194)
Sex	0.249					0.286
	(0.304)					(0.239)
Market distance	–0.043	0.198	–0.255	–0.393^**^	0.408	–0.330^**^
	(0.170)	(0.243)	(0.261)	**(0.170)**	(0.296)	**(0.168)**
Household mobility	–0.376^**^	–0.398	0.020	–0.100	–0.270	–0.014
	**(0.162)**	(0.287)	(0.202)	(0.216)	(0.225)	(0.162)
Household size	0.055	0.249	0.249	–0.031	–0.124	0.168
	(0.158)	(0.221)	(0.222)	(0.157)	(0.248)	(0.157)
SES (PC1 score)	0.024	–0.205	0.073	0.068	0.291	–0.027
	(0.148)	(0.197)	(0.243)	(0.140)	(0.270)	(0.147)
Constant	–0.088	0.229	–0.257	–0.166	0.225	–0.174
	(0.204)	(0.280)	(0.255)	(0.206)	(0.293)	(0.186)
Observations	55	31	23	29	22	54
Log likelihood	–79.70	–118.73	–85.81	–114.35	–91.97	–75.73
Akaike Inf. Crit.	177.41	253.46	187.62	244.70	199.94	169.46
Bayesian Inf. Crit.	195.47	264.93	196.70	255.64	208.67	187.36

^**^ *P* < 0.05; ^***^P < 0.01.

Body fat percentage increased slightly, but significantly, with age for women (*β* = 0.24, SE = 0.11, *P* = 0.02), but not men (Tables 3 and 4). Despite this, body fat percentage remained low among women, with those under the age of 40 having a mean body fat percentage of 21.3% ± 6.7% compared to 23.9% ± 7.9% among women over 40. Interestingly, despite these changes in body fat, there was not a similar association between BMI and age, as BMI remained very low throughout adulthood for men and women (Table 2). In concordance with the presence of low BMI, there was a high prevalence of adult underweight among Daasanach. About 76.7% of men sampled between the ages of 18 and 39 years were found to be underweight, while 71.4% of men 40 years and older were underweight. Women aged 18–39 years of age were found to have an underweight prevalence of 55.8%, while women aged 40 years and older had an underweight prevalence of 59.5% (Table 2). Only about a third of adults sampled (35.1%) were found to have BMI values within the range of ‘normal weight’ (BMI = 18.5–24.9 kg/m^2^), while less than 3% of all adults sampled were found to be overweight (BMI ≥ 25.0 kg/m^2^).

### The association between market integration and lifestyle variation on health

**Figure 4.**
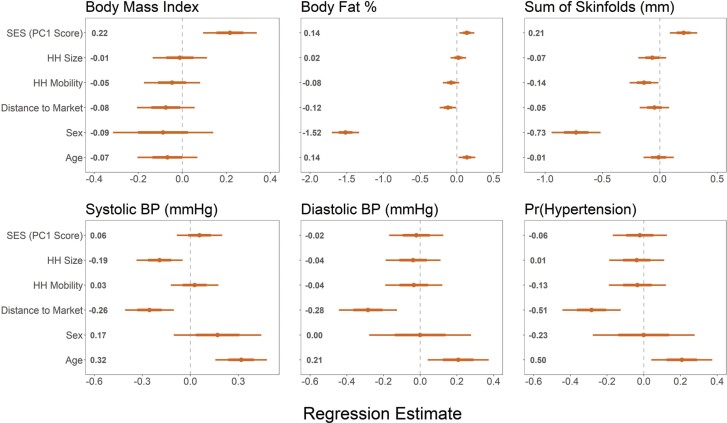
Anthropometric biomarkers of cardiometabolic health regression estimates for models of adults (>17 years). Points and numbers are regression estimates from linear mixed effects models with 75% CI (thick lines) and 95% CI (thin lines)

**Figure 5.**
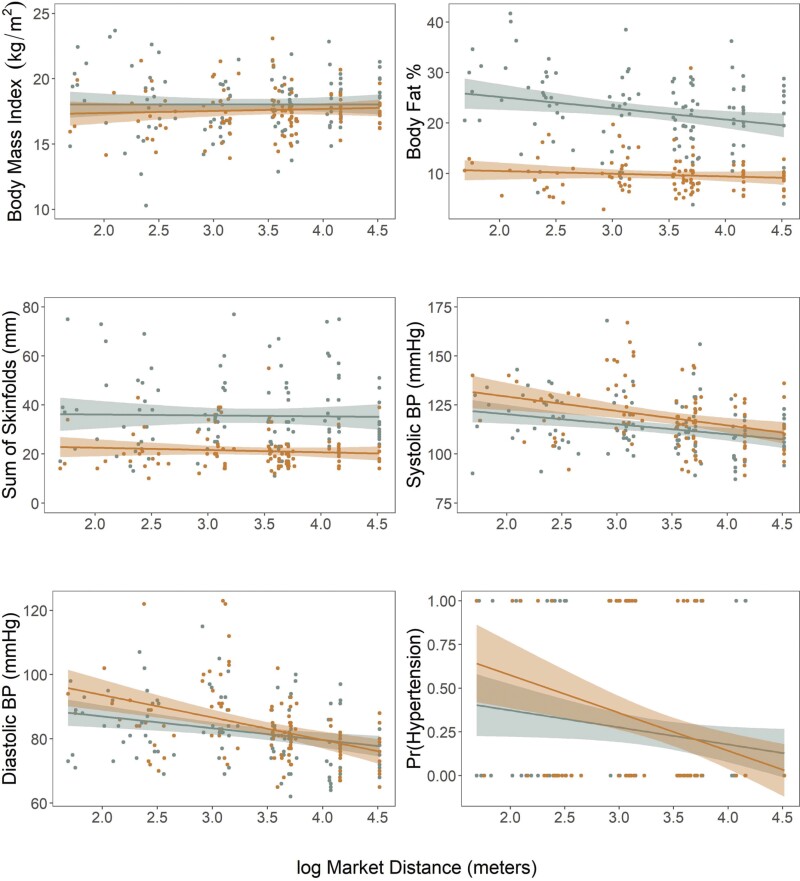
Relationships between anthropometric biomarkers of cardiometabolic health and market integration as measured by distance of household to the nearest market center in adults (>17 years; male = Orange; female = Blue)

To investigate the relationship between market integration and sedentarization on cardiometabolic health and body composition, multiple linear regression was used to test for the effects of lifestyle and socioecological variation on biomarkers for cardiometabolic health (Tables 3 and 4). Systolic and diastolic BP were both found to be significantly negatively correlated with household distance to the nearest market center (systolic: *β* = –0.26 mm Hg, SE = 0.08, *P* < 0.01 | diastolic: *β* = –0.28 mm Hg, SE = 0.08, *P* < 0.01) in adults (Figs. 4 and 5). To further investigate the effects of market integration on BP, the relationship between risk of hypertension and market distance was tested. Generalized logistic regression (Fig. 5) suggests 44% lower odds of having hypertension per unit of distance from a market center (OR = 0.56; 95% CI = 0.37–0.83, *P* < 0.01) controlling for age.

Unlike BP, body composition did not vary significantly with market distance among Daasanach adults. However, BMI was found to be significantly positively correlated with SES (*β* = 0.22, SE = 0.06, *P* < 0.01) across all adults (Fig. 4). Similarly, sum of skinfolds measures was found to increase with increased SES (Tables 3 and 4) for both men and women (female: *β* = 0.21, SE = 0.09, P = 0.03 | male: *β* = 0.18, SE = 0.06, *P* < 0.01), while body fat percentage, as measured by bioelectrical impedance, increased significantly with increased SES only among men (*β* = 0.10, SE = 0.04, *P* = 0.02). Women were found to have a similar trend for body fat percentage (*β* = 0.10, SE = 0.04, *P* = 0.02) but it was not significant. Household mobility was found to be negatively correlated with the sum of skinfold measures among men (Tables 3, 4 and Supplementary Fig. 2) such that men who reported moving more in the previous year had lower skinfold measures.

**Figure 6.**
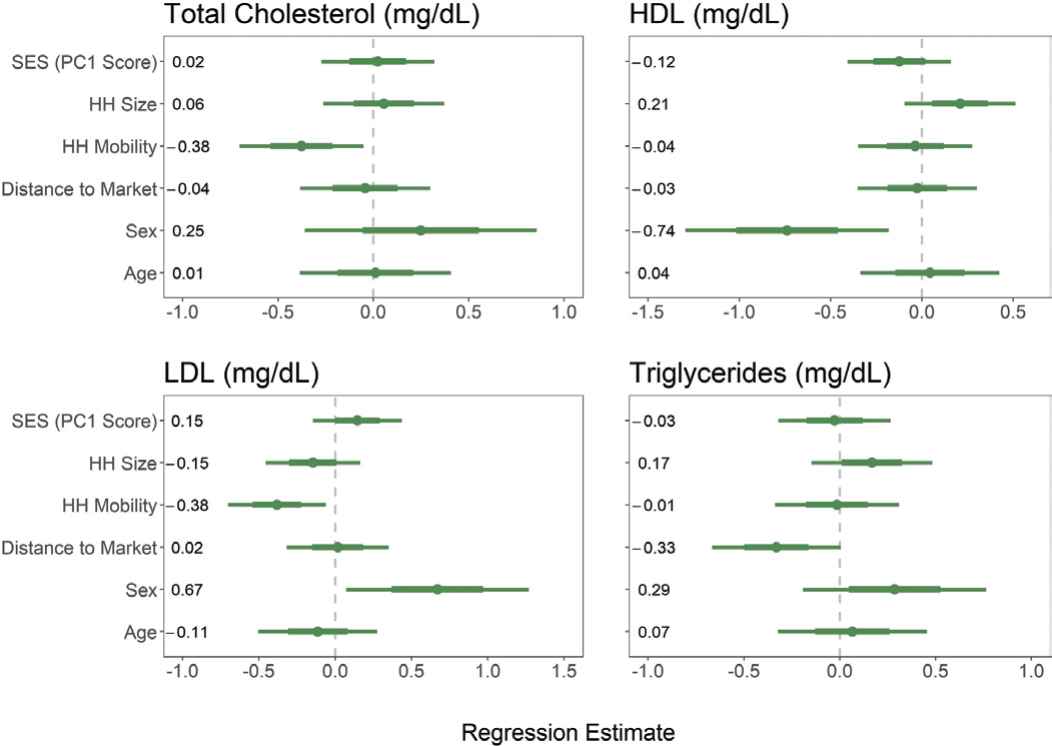
Blood chemistry biomarkers of cardiometabolic health regression estimates for models of adults (>17 years). Points and numbers are regression estimates from linear mixed effects models with 75% CI (thick lines) and 95% CI (thin lines)

For a subset of individuals, additional biomarkers were collected from blood chemistry via finger prick blood samples. LDL levels significantly decreased as distance from the market increased (*β* = –0.39, SE = 0.17, *P* = 0.03) among women (Table 1). Across both men and women, triglyceride levels were also significantly negatively associated with distance to the nearest market (*β* = –0.33, SE = 0.17, *P* = 0.05; Tables 3, 4 and Fig. 6). Total cholesterol levels were negatively correlated with household mobility, with individuals reporting a larger number of household moves in the previous year having significantly lower cholesterol (*β* = –0.38, SE = 0.16, *P* = 0.03). HDL values did not vary by any lifestyle measure (Fig. 6).

## DISCUSSION

This study sought to test the effects of lifestyle change on biomarkers of NCD health. The effects of lifestyle change, namely market integration (as measured by a household’s distance to the nearest market) and sedentarization (as measured by the number of times a household moved in the previous year), on biomarkers for cardiometabolic health were large enough to detect across subjects. Results suggest heterogeneous effects on health measures as they relate to lifestyle characteristics.

First, systolic and diastolic BP in adults both decreased as a function of market distance when controlling for age, as well as the probability of hypertension. These associations match expectations based on previous work that has found that individuals living in small-scale subsistence societies who are less market integrated tend to have lower BP [3, 5, 15, 27]. For Daasanach, results are also influenced by environmental factors, as recent work found that groundwater nearer to Illeret contains higher levels of salt and there is access to water with higher salinity levels, which likely contributes to increased hypertension risk for more settled and market-integrated communities [41]. However, like results found in previous work with small-scale populations living in developing nations, Daasanach show hypertension rates that are markedly lower than those of adult US men and women, as hypertension affects nearly half of American adults (men: 50.6 ± 1.2%, women: 45.7 ± 1.5%) [6, 7, 13, 44, 49, 50].

While previous work has found significant differences in BMI across market-integrated and non-market-integrated, or urban and rural individuals, market distance and household mobility were not associated with BMI for adults in our sample [3, 6, 23]. BMI for men and women was found to be very low across our sample, with a majority of adults having a BMI < 18.5 kg/m^2^. However, despite the uniformly low measured BMI values, BMI was found to be positively correlated with SES score. Greater market distance and mobility were associated with greater food insecurity, yet our prior work has shown that ~98% of Daasanach are moderate to severely food insecure [40]. Thus, even those living a more sedentarized lifestyle in Illeret suffer from food insecurity and thus this food insecurity may blunt effects of lifestyle change on changes in adiposity. Additionally, skinfold measures were also found to positively correlate with SES in men and women, while body fat percentage, as measured by bioelectrical impedance, significantly increased with increased SES among men. This could indicate the nutritional role of livestock wealth, with greater animal ownership leading to more milk and calories from animals. Our prior work found that milk consumption among Daasanach had cardioprotective benefits [41]. These results match recent findings from work with the Turkana of Kenya, which suggested that socioeconomic status predicted increased BMI and body fat in urban and rural populations [51]. The results found with Daasanach adults also match expectations based on previous work with Eastern African pastoralist populations, which found that body fat, as measured by skinfolds, was significantly higher among more market-integrated and settled women [2, 4, 23], though these studies did not explicitly investigate socioeconomic condition.

Additional biomarkers of health were collected from blood chemistry measured from finger prick blood sampling. Like previously discussed anthropometric measures, blood lipids were also found to differ with variations in lifestyle characteristics. Total cholesterol was significantly lower among adults who were engaged in more traditional nomadic behavior through increased household mobility, and triglyceride levels were significantly lower among those who lived farther from market centers. Linear mixed effects models found that both HDL and LDL levels have a significant sex interaction in our sample, thus men and women were modeled separately. In doing so, no lifestyle variables were found to significantly correlate for HDL in either men or women, and no significant correlations were identified for LDL in men. Like triglycerides, however, LDL levels were significantly lower in women who lived farther from market centers. These findings in blood chemistry variation generally match those across anthropometric measures of cardiometabolic health, particularly as they apply to BP, though there was lower power to detect associations with a smaller sample size. Further investigation is needed to understand the sex-specific differences in the relationship between market integration and LDL levels.

While several biomarkers for NCD health were found to vary as a function of lifestyle change, the association of market integration and mobility on nutritional, behavioral and socioeconomic variation could also be assessed. Previous work with Maasai pastoralists found that nutritional status remained poor despite increased market integration and level of sedentarization [52]. Food and water insecurity were both negatively correlated with proxies for market integration and sedentarization, but the overall prevalence of insecurity remained very high across the entire sample [39]. Similarly, levels of educational achievement were higher among those who were closer to market centers and more settled, but educational achievement was low across both men and women, with most subjects having never attended any level of schooling.

There is some indication that variation in market integration and engagement in traditional pastoral activities do affect nutrition beyond levels of nutritional security. Typically, African pastoralists have diets that are high in protein, low in fat, with relatively little access to sugar [3, 52, 53]. Self-reported meat consumption did not vary across our sample. Our team’s observations of increased frequency of consuming fish, which is not part of the traditional Daasanach diet, can result in a significantly higher rate of protein consumption overall in those nearer to market centers. This has occurred recently as livestock have died due to severe drought. Increased access to fish has potentially significant nutritional and health consequences among Daasanach, who already suffer from high levels of malnutrition, as expectations based on previous work with East African pastoralists would be for decreased protein consumption with increased market integration and sedentarization [4]. Access to Lake Turkana and fishing may buffer some of the nutritional effects of lifestyle change.

Though variation in body composition, cardiometabolic condition and lifestyle across Daasanach living in northern Kenya is suggestive of the effects of evolutionary mismatch, determinations about the detrimental effects of lifestyle change on Daasanach at a population level are more difficult to assess. Typical biomarkers for cardiometabolic disease, like BMI, body fat percentage and BP are all low across adult Daasanach in our sample. While previous work has suggested that sedentarization has negative effects on nutritional, reproductive and health outcomes, the findings here represent trends toward expected outcomes, but not explicit variation in cardiometabolic disease outcomes themselves [2, 14, 54–58]. For example, within industrialized contexts, increases in BMI and body fat are often biomarkers of poor health [59]. However, among Daasanach, increased BMI and body fat percentage are likely indicators of increased health conditions as most adults are clinically underweight and have low body fat percentages relative to Western averages [44, 60, 61].

There are several limitations of this study. First, adult age was estimated when the exact age was unknown and could not be verified, which can lead to error within the linear mixed effects models that control for age as a fixed effect. To reduce the effect of age estimation, all ages were estimated with the aid of a known historical event calendar from which a local translator and research assistant were then able to confirm a subject’s age in years. In addition, the measurement of SES, which is often understood within an industrialized context, requires a specific cultural context when assessed within the Daasanach population [62]. For this reason, three culturally relevant measures (i.e. income, perception of socioeconomic standing and livestock holdings) were used to create a composite score of Daasanach-specific SES values. These values are, therefore, not comparable to ‘Western’ measures of SES or across other small-scale populations. Additionally, blood lipid models found no significance in our analyses of SES, despite the significant association between SES and body composition. This result may be due to the relatively low sample size for blood chemistry analyses, which were limited by the time of data collection and subject condition. Also, while food and water insecurity and nutritional data were collected during household surveys, robust collection of dietary data did not occur for the purposes of this study. Therefore, models investigating the relationships between variations in daily dietary intake, lifestyle and biomarkers of health were not available. Future work will include increased sensitivity of dietary data collection, as well as increased sample sizes and community diversity to bolster biomarker models.

Populations like Daasanach in northern Kenya, in the nascent stages of lifestyle transition, provide an important opportunity to investigate heterogeneous effects of early market integration and sedentarization on NCD health. Such work is essential to understand the complex dynamics underlying the effects of these trends on health and well-being as communities like the Daasanach continue to move away from traditional semi-nomadic pastoral practices. Understanding the relationships between cardiometabolic health and lifestyle will advance global public health strategies as the pace of change accelerates in response to external and internal market forces as well as climatic stressors.

## Supplementary Material

eoad030_suppl_Supplementary_Figure_1

eoad030_suppl_Supplementary_Figure_2
