# Supplementary figures and images for: The effects of lifestyle change on indicators of cardiometabolic health in semi-nomadic pastoralists
Source: Evol Med Public Health. 2023 Sep 25;11(1):318–31. doi: 10.1093/emph/eoad030 (PMC10576223; doi:10.1093/emph/eoad030)

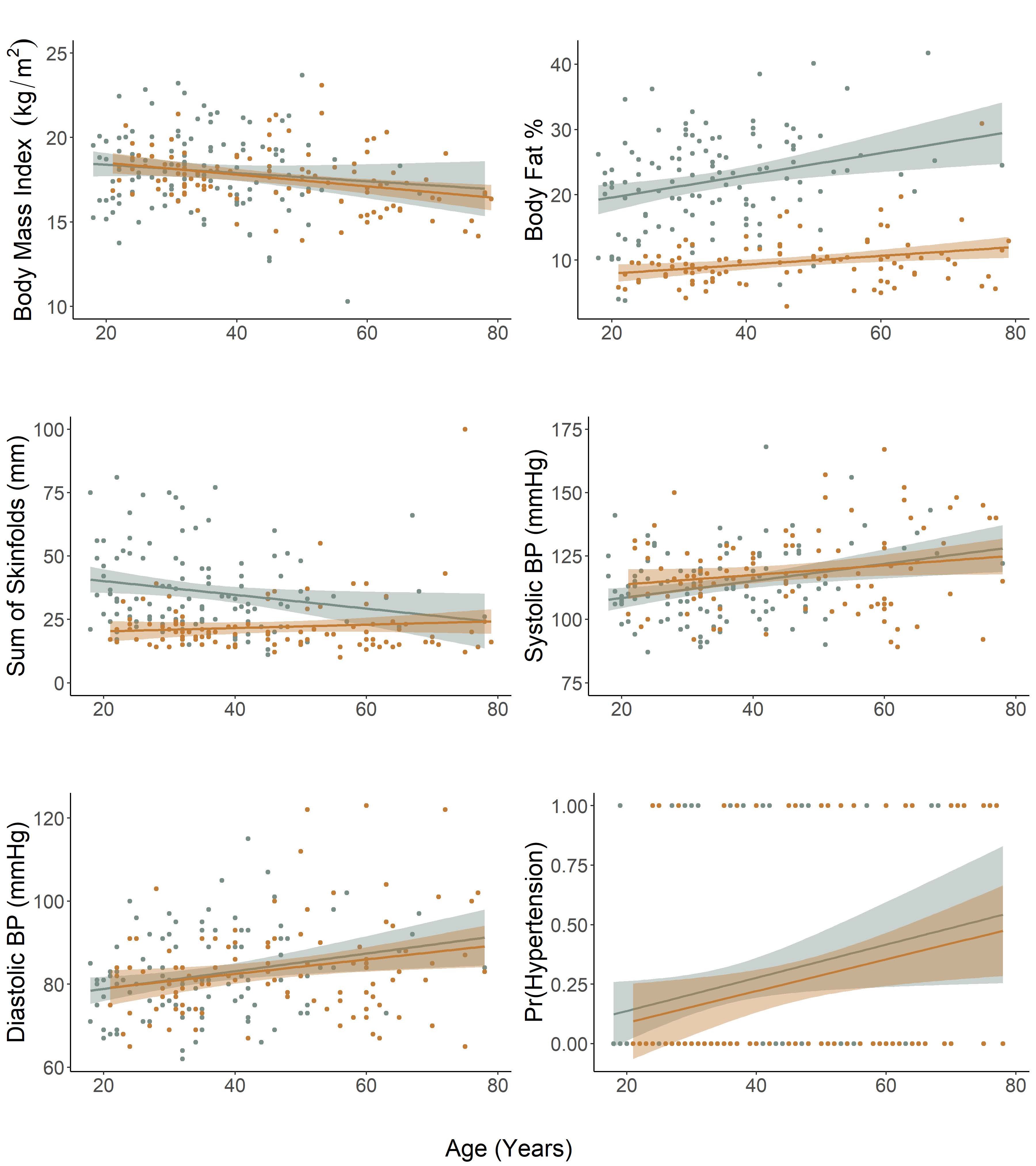

Supplement: eoad030_suppl_Supplementary_Figure_1 [file eoad030_suppl_supplementary_figure_1.jpeg]

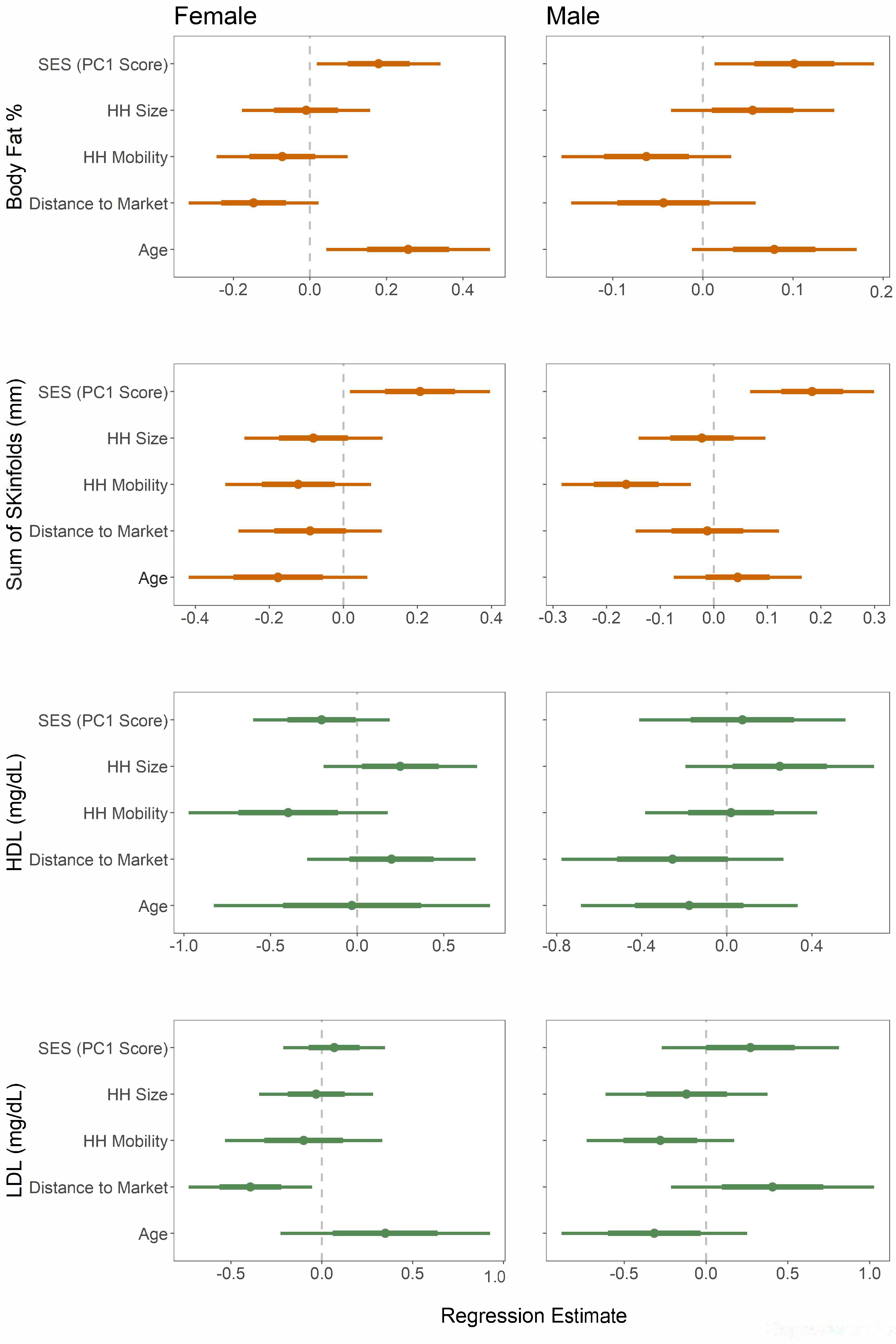

Supplement: eoad030_suppl_Supplementary_Figure_2 [file eoad030_suppl_supplementary_figure_2.jpeg]
